# Supplementary material for: Iterative improvement in the automatic modular design of robot swarms
Source: PeerJ Comput Sci. 2020 Dec 7;6:e322. doi: 10.7717/peerj-cs.322 (PMC7924708; doi:10.7717/peerj-cs.322)
Supplement: Supplemental Information 3 [file peerj-cs-06-322-s003.zip › argos3/doc/api/standalone/a00425_source.html]

ARGoS: plugins/robots/eye-bot/simulator/eyebot\_entity.h Source File


- Main Page
- Related Pages
- Namespaces
- Classes
- Files

- File List
- File Members

# plugins/robots/eye-bot/simulator/eyebot\_entity.h

Go to the documentation of this file.

```
00001 
00007 #ifndef EYEBOT_ENTITY_H
00008 #define EYEBOT_ENTITY_H
00009 
00010 namespace argos {
00011    class CControllableEntity;
00012    class CEmbodiedEntity;
00013    class CEyeBotEntity;
00014    class CLEDEquippedEntity;
00015    class CLightSensorEquippedEntity;
00016    class CPerspectiveCameraEquippedEntity;
00017    class CProximitySensorEquippedEntity;
00018    class CQuadRotorEntity;
00019    class CRABEquippedEntity;
00020 }
00021 
00022 #include <argos3/core/simulator/entity/composable_entity.h>
00023 
00024 namespace argos {
00025 
00026    class CEyeBotEntity : public CComposableEntity {
00027 
00028    public:
00029 
00030       ENABLE_VTABLE();
00031 
00032    public:
00033 
00034       CEyeBotEntity();
00035 
00036       CEyeBotEntity(const std::string& str_id,
00037                     const std::string& str_controller_id,
00038                     const CVector3& c_position = CVector3(),
00039                     const CQuaternion& c_orientation = CQuaternion(),
00040                     Real f_rab_range = 3.0f,
00041                     size_t un_rab_data_size = 10,
00042                     const CRadians& c_perspcam_aperture = ToRadians(CDegrees(30.0f)),
00043                     Real f_perspcam_focal_length = 0.035,
00044                     Real f_perspcam_range = 2.0);
00045 
00046       virtual void Init(TConfigurationNode& t_tree);
00047       virtual void Reset();
00048 
00049       virtual void UpdateComponents();
00050       
00051       inline CControllableEntity& GetControllableEntity() {
00052          return *m_pcControllableEntity;
00053       }
00054 
00055       inline CEmbodiedEntity& GetEmbodiedEntity() {
00056          return *m_pcEmbodiedEntity;
00057       }
00058 
00059       inline CLEDEquippedEntity& GetLEDEquippedEntity() {
00060          return *m_pcLEDEquippedEntity;
00061       }
00062 
00063       inline CLightSensorEquippedEntity& GetLightSensorEquippedEntity() {
00064          return *m_pcLightSensorEquippedEntity;
00065       }
00066 
00067       inline CProximitySensorEquippedEntity& GetProximitySensorEquippedEntity() {
00068          return *m_pcProximitySensorEquippedEntity;
00069       }
00070 
00071       inline CQuadRotorEntity& GetQuadRotorEntity() {
00072          return *m_pcQuadRotorEntity;
00073       }
00074 
00075       inline CRABEquippedEntity& GetRABEquippedEntity() {
00076          return *m_pcRABEquippedEntity;
00077       }
00078 
00079       virtual std::string GetTypeDescription() const {
00080          return "eye-bot";
00081       }
00082 
00083    private:
00084 
00085       CControllableEntity*              m_pcControllableEntity;
00086       CEmbodiedEntity*                  m_pcEmbodiedEntity;
00087       CLEDEquippedEntity*               m_pcLEDEquippedEntity;
00088       CLightSensorEquippedEntity*       m_pcLightSensorEquippedEntity;
00089       CPerspectiveCameraEquippedEntity* m_pcPerspectiveCameraEquippedEntity;
00090       CProximitySensorEquippedEntity*   m_pcProximitySensorEquippedEntity;
00091       CQuadRotorEntity*                 m_pcQuadRotorEntity;
00092       CRABEquippedEntity*               m_pcRABEquippedEntity;
00093    };
00094 
00095 }
00096 
00097 #endif
```

---

Generated on 10 Jul 2018 for ARGoS by 
 1.6.1
